# CelloType: a unified model for segmentation and classification of tissue images

**DOI:** 10.1038/s41592-024-02513-1

**Published:** 2024-11-22

**Authors:** Minxing Pang, Tarun Kanti Roy, Xiaodong Wu, Kai Tan

**Affiliations:** 1 Applied Mathematics and Computational Science Graduate Group, University of Pennsylvania, Philadelphia, PA USA; 2 Department of Computer Science, University of Iowa, Iowa City, IA USA; 3 Department of Electrical and Computer Engineering, University of Iowa, Iowa City, IA USA; 4 Department of Radiation Oncology, University of Iowa, Iowa City, IA USA; 5 Department of Pediatrics, Perelman School of Medicine, University of Pennsylvania, Philadelphia, PA USA; 6 Division of Oncology and Center for Childhood Cancer Research, Children’s Hospital of Philadelphia, Philadelphia, PA USA; 7 Center for Single Cell Biology, Children’s Hospital of Philadelphia, Philadelphia, PA USA

**Keywords:** Image processing, Machine learning, Software

## Abstract

Cell segmentation and classification are critical tasks in spatial omics data analysis. Here we introduce CelloType, an end-to-end model designed for cell segmentation and classification for image-based spatial omics data. Unlike the traditional two-stage approach of segmentation followed by classification, CelloType adopts a multitask learning strategy that integrates these tasks, simultaneously enhancing the performance of both. CelloType leverages transformer-based deep learning techniques for improved accuracy in object detection, segmentation and classification. It outperforms existing segmentation methods on a variety of multiplexed fluorescence and spatial transcriptomic images. In terms of cell type classification, CelloType surpasses a model composed of state-of-the-art methods for individual tasks and a high-performance instance segmentation model. Using multiplexed tissue images, we further demonstrate the utility of CelloType for multiscale segmentation and classification of both cellular and noncellular elements in a tissue. The enhanced accuracy and multitask learning ability of CelloType facilitate automated annotation of rapidly growing spatial omics data.

## Abstract (web summary)

CelloType is an end-to-end method for spatial omics data analysis that uses a transformer-based deep neural network for concurrent object detection, segmentation and classification and performs with high accuracy on diverse datasets.

## Main

Recent advancements in spatial omics technologies have markedly improved our ability to analyze intact tissues at the cellular level, revealing unparalleled insights into the link between cellular architecture and functionality of various tissues and organs^1^. Collaborative efforts, such as the Human Tumor Atlas Network^2^, the Human Biomolecular Atlas Program^3^ and the BRAIN initiative, are leveraging these technologies to map spatial organizations of various types of healthy and diseased tissue. With the anticipated surge in spatial omics data, there is a pressing need for sophisticated computational tools for data analysis. A typical analysis workflow of spatial omics data begins with cell segmentation. Following cell segmentation and quantification of molecular analytes, cell type annotation is the next critical, albeit often time-consuming, task before further analysis can proceed. Conventional analysis pipelines perform these two tasks sequentially, typically using the segmentation results as the inputs for the classification task. As representatives of state-of-the-art segmentation methods, Mesmer^4^ uses a convolutional neural network (CNN)^5^ backbone and a feature pyramid network with the watershed algorithm for both nuclear and cell segmentation. Cellpose^6^ and Cellpose2^7^ use a CNN with a U-net^8^ architecture to predict the gradient of topological map. A gradient tracking algorithm is then used to obtain the segmentation mask. For the cell classification task, CellSighter^9^ employs CNN to predict cell types on the basis of segmentation masks and the tissue images. CELESTA^10^ uses an iterative algorithm to assign cell types on the basis of a quantified cell-by-protein matrix.

Despite achieving satisfactory performance in certain tissues, conventional approaches have several limitations. First and foremost, the reliance of cell classification models on segmentation results hampers their ability to leverage the full spectrum of semantic information present in tissue images. In fact, these two tasks are interconnected. Segmentation can enhance focus on relevant signals, thus mitigating noise and enabling more precise learning of class features for classification. Conversely, information specific to classes aid in the segmentation process, as the unique texture and morphology of certain object types can enhance segmentation accuracy. Second, the two-step approach is computationally inefficient, requiring separate training for each task. Third, the performance of existing segmentation methods also varies significantly across different tissue types, suggesting substantial room for improvement. Moreover, to our knowledge, existing methods do not offer a confidence assessment for the segmentation task.

Deep learning, especially through the use of CNNs, has gained popularity in biomedical image analysis, especially in segmentation^11^ and classification^9^. Mesmer, for example, has notably improved cell segmentation accuracy using CNN. Unlike Mesmer which does semantic segmentation, mask region-based CNN (Mask R-CNN) models can perform instance segmentation with improved accuracy^12^. However, recent developments in computer vision have shown that transformer-based models^13^, such as the detection transformer (DETR)^14^ and the detection transformer with improved denoising anchor (DINO)^15^, significantly outperform CNN-based models in object detection. These transformer-based models have also shown superior performance in instance segmentation of histological images^16^. Despite these breakthroughs, the application of transformer-based models to cell/nuclear segmentation in multiplexed images and other spatial omics data type remains unexplored. A unified framework, MaskDINO^17^, which integrates object detection and segmentation, has shown superior performance across diverse datasets for multiclass instance segmentation. However, its effectiveness has only been tested on three-channel Red, Green, and Blue (RGB) images of natural objects. This leaves a considerable gap in applying transformer-based models to multiplexed tissue images, which present greater challenges due to their larger number of imaging channels, varying shapes of tightly apposed/overlapping cellular and noncellular elements.

The limitations of current methodologies and the advent of novel deep learning techniques motivated us to develop CelloType, an end-to-end method for joint cell segmentation and classification. CelloType employs a transformer-based deep neural network (DNN) architecture with multiple branches to handle object detection, segmentation and classification concurrently. We benchmarked the performance of CelloType against state-of-the-art methods using a variety of public image datasets, including single-channel and multiplexed fluorescence tissue and cell images and bright-field images of nature objects. We further demonstrated a novel feature of CelloType for multiscale segmentation and classification to delineate both cellular and noncellular elements in tissue images.

## Results

### Overview of CelloType

**Fig. 1:**
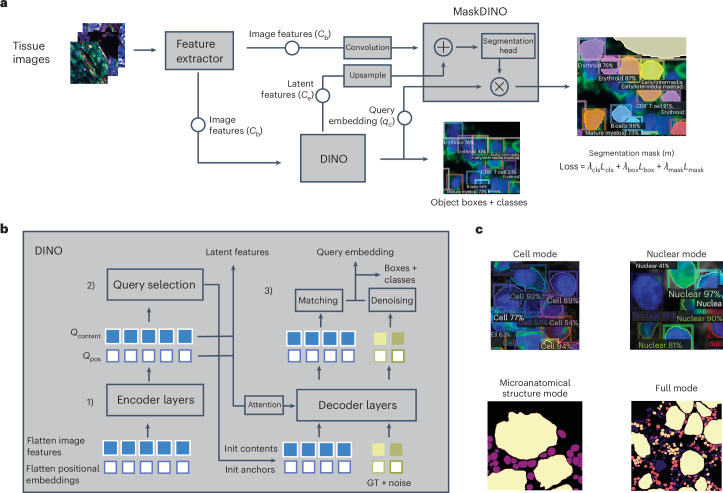
Overview of CelloType. **a**, The overall architecture, input and output of CelloType. First, a Transformer-based feature extractor is employed to derive multiscale features (*C*_b_) from the image. Second, using a Transformer-based architecture, the DINO object detection module extracts latent features (*C*_e_) and query embeddings (*q*_c_) that are combined to generate object detection boxes with cell type labels. Subsequently, the MaskDINO module integrates the extracted image features with DINO’s outputs, resulting in detailed instance segmentation and cell type classification. During training, the model is optimized on the basis of an overall loss function (Loss) that considers losses based on cell segmentation mask (*λ*_mask_*L*_mask_), bounding box (*λ*_box_*L*_box_) and cell type label (*λ*_cls_*L*_cls_). **b**, The input, output and architecture of the DINO module. The DINO module consists of a multilayer transformer and multiple prediction heads. DINO starts by flattening the multiscale features from the transformer-based feature extractor. These features are merged with positional embeddings to preserve spatial context (step 1). DINO then employs a mixed query selection strategy, initializing positional queries (*Q*_pos_) as anchor detection boxes and maintaining content queries (*Q*_content_) as learnable features, thus adapting to the diverse characteristics of cells (step 2). The model refines these anchor boxes through decoder layers using deformable attention mechanism and employs contrastive denoising training by introducing noise to ground-truth (GT) labels and boxes to improve robustness and accuracy. Then, a linear projection acts as the classification branch to produce the classification results for each box (step 3). **c**, The multiscale ability of CelloType. CelloType is versatile and can perform a range of end-to-end tasks at different scales, including cell segmentation, nuclear segmentation, microanatomical structure segmentation and full instance segmentation with corresponding class annotations.

CelloType is a DNN-based framework (Fig. 1) designed for joint multiscale segmentation and classification of a variety of biomedical microscopy images, including multiplexed molecular images, histological images and bright-field images. The core of CelloType’s functionality begins with the extraction of multiscale image features through the use of Swin Transformer^18^. These features are then fed into the DINO object detection module that extracts instance-specific latent features and predicts a preliminary object bounding box with an associated class label for each instance. Finally, the MaskDINO segmentation module integrates the multiscale image features from Swin Transformer and DINO outputs to produce the final refined instance segmentations. The CelloType model is trained using a loss function that considers segmentation masks, object detection boxes and class labels.

The DINO module’s architecture (Fig. 1b) includes a transformer encoder–decoder setup with multiple prediction heads. It begins by flattening image features and integrating them with positional embeddings^19^. By employing a strategy that mixes anchor and content queries, the module can adapt to various object features. The module refines bounding boxes through a deformable attention mechanism. A contrastive denoising training procedure is used together with the attention mechanism to improve the robustness of bounding box detection. Finally, a linear transformation is applied to the denoised bounding box features to predict the class label of the object.

CelloType can tackle diverse image analysis tasks including cell/nuclear segmentation, noncellular structure segmentation and multiscale segmentation (Fig. 1c). Different data types are used to train CelloType for various tasks. For cell or nuclear segmentation, training data include one- or two-channel images with corresponding cell membrane or nuclear masks. For joint segmentation and classification, the training data consist of images with segmentation mask, bounding box and class label of each object. The images can contain many channels in addition to the cell membrane and nuclear channels. CelloType is implemented in Python and publicly available via GitHub at http://github.com/tanlabcode/CelloType.

### Benchmark of cell segmentation on multiplexed images

We first applied CelloType to the TissueNet dataset^4^ that includes tissue images generated using six multiplexed molecular imaging technologies (codetection by indexing (CODEX)^20^, cyclic immunofluorescence (CycIF)^21^, imaging mass cytometry (IMC)^22^, multiplexed ion beam imaging (MIBI)^23^, multiplexed immunofluorescence (MxIF)^24^ and Vectra^25^) and six tissue types (breast, gastrointestinal, immune, lung, pancreas and skin). The images were divided into 2,580 training patches (512 × 512 pixels) and 1,324 test patches (256 × 256 pixels).

**Fig. 2:**
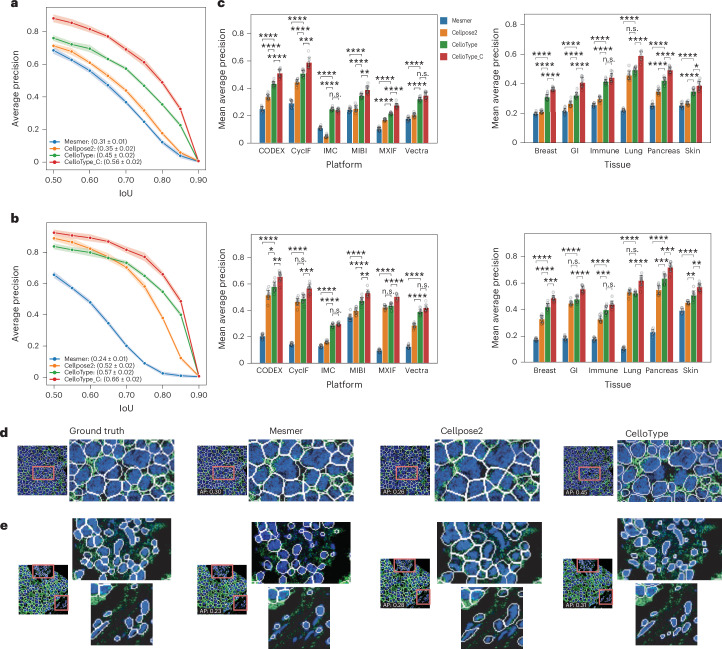
Evaluation of segmentation accuracy using TissueNet datasets. **a**, A line plot showing AP across IoU thresholds for cell segmentation by Mesmer, Cellpose2, CelloType and CelloType_C (CelloType with confidence score). Each data point represents the average AP from a 10-fold cross-validation experiment. Band width around each line represents the standard deviation. The mean and standard deviation of average AP values across IoU thresholds are shown in the parentheses. **b**, AP across IoU thresholds for nuclear segmentation. The band width around each line represents the standard deviation. **c**, The performance of methods stratified by imaging platform and tissue type. Each grouped barplot is overlaid with ten data points, representing the results of a 10-fold cross-validation. The error bar represents the standard deviation. The top left grouped barplot shows mean AP values for cell segmentation stratified by imaging platform, including CODEX, CyCIF, IMC, MIBI, MxIF and Vertra. The top right grouped barplot shows mean AP values for cell segmentation stratified by tissue type, including breast, gastrointestinal, immune, pancreas and skin. The second row of grouped barplots shows mean AP values for nuclear segmentation. Statistical significance is indicated as follows: ****P < 1 × 10^−4^, ***P < 1 × 10^−3^, **P < 1 × 10^−2^, *P < 0.05. *P* values were computed using one-sided Student’s *t*-test (Supplementary Tables 5–8). **d**, Representative examples of cell segmentation of immune tissue imaged using Vectra platform. Blue, nuclear signal; green, cell membrane signal; white, cell boundary. The red box highlights a representative region that the methods perform differently. A zoomed-in view of the highlighted image area is shown to the right of the full image. The image-level AP scores are shown on the images. **e**, Representative examples of nuclear segmentation of gastrointestinal tissue using the IMC platform.

**Extended Data Fig. 1:**
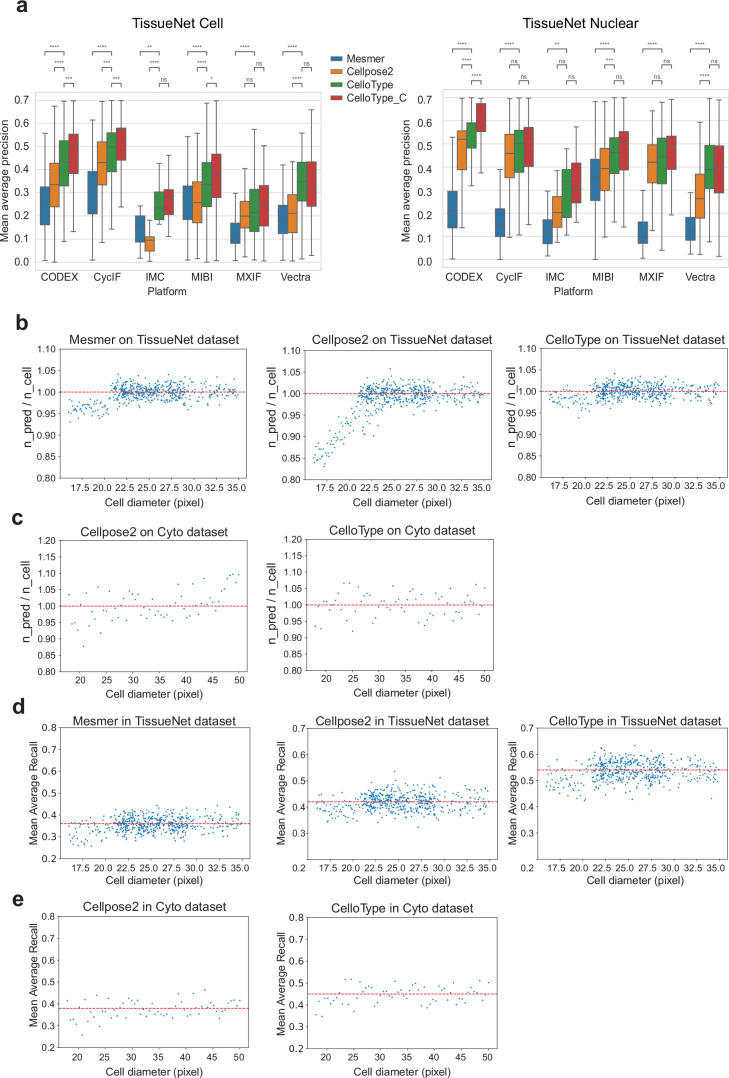
Image level segmentation performance of compared methods. **a**) Boxplots of image-level mean average precision (AP) for cell segmentation (left panel) and nuclear segmentation (right panel) stratified by experimental platforms. The box represents the interquartile range (IQR), with its lower bound indicating the 25th percentile and the upper bound indicating the 75th percentile. Whiskers extend from the box to show the range of the data, with the lower whisker extending to the minimum value (or to the smallest data point within 1.5 times the IQR) and the upper whisker extending to the maximum value (or to the largest data point within 1.5 times the IQR). The number of images in the test dataset for each platform is as following: CODEX (397), CycIF (322), IMC (16), MIBI (248), MxIF (87), and Vectra (179). Statistical significance is indicated as following: **** p-value < 1e-4, *** p-value < 1e-3, ** p-value < 1e-2, * p-value < 0.05. P-valued were computed using one-sided Student’s t-test (Supplementary Table 5). **b-c**) Scatter plots of image-level relationship between cell size and segmentation accuracy. Each data point represents an image. X-axis, average diameter of cells in an image. Y-axis, ratio of the number of predicted cells to the number of true cells in the dataset (n_pred/n_cell). **d-e**) Scatter plots of image-level relationship between cell size and segmentation recall rate. Each data point represents an image. X-axis, average diameter of cells in an image. Y-axis, mean recall rate across the IoU threshold range (0.5–0.9).

We compared CelloType with two state-of-the-art methods, Mesmer^4^ and Cellpose2^7^. For object detection and segmentation, we used the average precision (AP) metric^26^ defined by the Common Objects in Context (COCO) project and the intersection over union (IoU) thresholds from 0.5 to 0.9 in 0.05 increments (Methods). The AP–IoU curves based on 10-fold cross-validation revealed that CelloType consistently outperformed both Mesmer and Cellpose2 across the entire range of IoU thresholds on the TissueNet dataset (Fig. 2a and Supplementary Table 1). In addition, considering that CelloType provides a confidence score for each segmentation mask and the COCO metric incorporates these confidence scores in matching predicted and ground-truth cell boundaries, we also evaluated a version of CelloType that outputs confidence scores, CelloType_C. Overall, performance is higher for cell segmentation than nuclear segmentation for all methods except for Mesmer. For cell segmentation, CelloType_C achieved a mean AP of 0.56, significantly surpassing the basic CelloType (0.45), Cellpose2 (0.35) and Mesmer (0.31) (Fig. 2a and Supplementary Table 1). For nuclear segmentation, CelloType_C achieved a mean AP of 0.66, outperforming CelloType (0.57), Cellpose2 (0.52) and Mesmer (0.24) by considerable margins (Fig. 2b and Supplementary Table 2). These results underscore CelloType’s superior segmentation accuracy and the added value of confidence scores. We also confirmed the superior performance of CelloType at the individual image level (Extended Data Fig. 1a and Supplementary Tables 3 and 4).

To evaluate the effect of imaging technology and tissue type on the segmentation performance, we next analyzed the mean AP scores stratified by these two factors (Fig. 2c and Supplementary Tables 5–8). Overall, performance of all methods is lowest on the IMC data and breast tissue data. CelloType and CelloType_C consistently outperformed Mesmer and Cellpose2 across the technology platforms and tissue types. Figure 2d,e shows representative cell and nuclear segmentation results by the compared methods. These examples illustrate that Cellpose2 tends to produce segmentation boundaries that are larger than the ground truth, resulting in undersegmentation (Extended Data Fig. 1b,c). On the other hand, Mesmer tends to miss more cells or nuclei as measured by the recall rate (Extended Data Fig. 1d,e).

### Benchmark of cell segmentation using diverse image types

To further evaluate CelloType’s performance of cell segmentation across diverse microscopy images beyond multiplexed fluorescence images, we applied CelloType to the Cellpose Cyto dataset^6^, which include fluorescence, bright-field microscopy images of cells and images of natural objects. Since most of the images in this dataset contain only one channel and Mesmer was trained on two-channel image data, we only benchmarked the performance of CelloType, CelloType_C and Cellpose2.

**Fig. 3:**
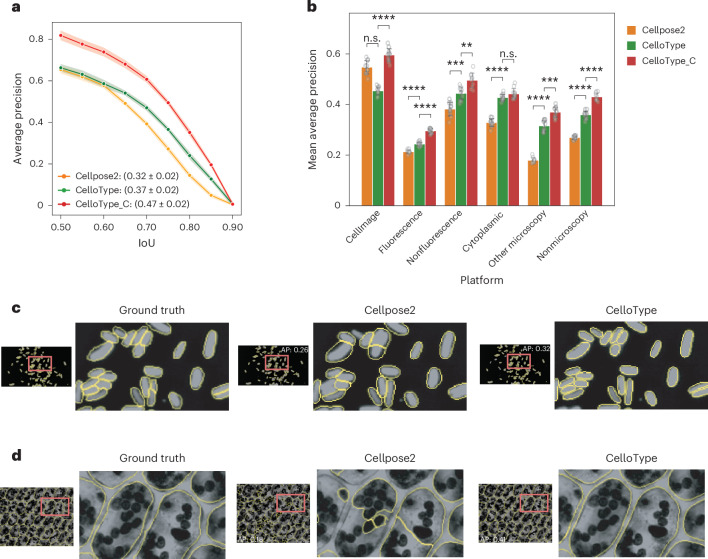
Evaluation of segmentation accuracy using the Cellpose Cyto dataset. **a**, A line plot showing AP across IoU thresholds for cell segmentation for Cellpose2, CelloType and CelloType_C (CelloType with confidence score). Each data point represents the average AP from a 10-fold cross-validation experiment. The band width around each line represents the standard deviation. The mean and standard deviation of average AP values across IoU thresholds are shown in the parentheses. **b**, The performance of methods stratified by image type. The mean AP values of Cellpose2, CelloType and CelloType_C are stratified by imaging modality and cell type. Each grouped barplot is overlaid with ten data points, representing the results of a 10-fold cross-validation. The error bar represents the standard deviation. The test dataset comprises microscopy and nonmicroscopy images from the Cellpose Cyto dataset that comprises six subsets, including cells (Cell Image Library), cells (fluorescence), cells (nonfluorescence), cells (membrane), other microscopy and nonmicroscopy. Statistical significance is indicated as follows: ****P < 1 × 10^−4^, ***P < 1 × 10^−3^, **P < 1 × 10^−2^, *P < 0.05. *P* values were computed using one-sided Student’s *t*-test (Supplementary Table 10). **c**, Representative examples of cell segmentation of a microscopy image by the compared methods. The red boxes highlight a representative region that the methods perform differently. A zoomed-in view of the highlighted image area is shown to the right of the full image. Image-level AP scores are shown on the images. **d**, Representative examples of cell segmentation of a nonfluorescence image by the compared methods.

Across the entire dataset, CelloType_C achieved a mean AP of 0.47, surpassing the performance of both CelloType (0.37) and Cellpose2 (0.32) based on 10-fold cross-validation (Fig. 3a and Supplementary Table 9). This superiority is consistently observed across six diverse image datasets (Fig. 3b and Supplementary Table 10). Figure 3c shows representative segmentation results by Cellpose2 and CelloType for a single-channel image from the ‘other microscopy’ category. Consistent with the findings in Fig. 2d with multiplexed IMC image, Cellpose2 exhibited a tendency for undersegmentation, while CelloType produced more precise segmentation boundaries. In addition, Fig. 3d shows the segmentation result for another single-channel image from the ‘nonfluorescence’ cell category, where CelloType demonstrated enhanced accuracy in both identifying the correct number of cells and delineating their boundaries.

**Extended Data Fig. 2:**
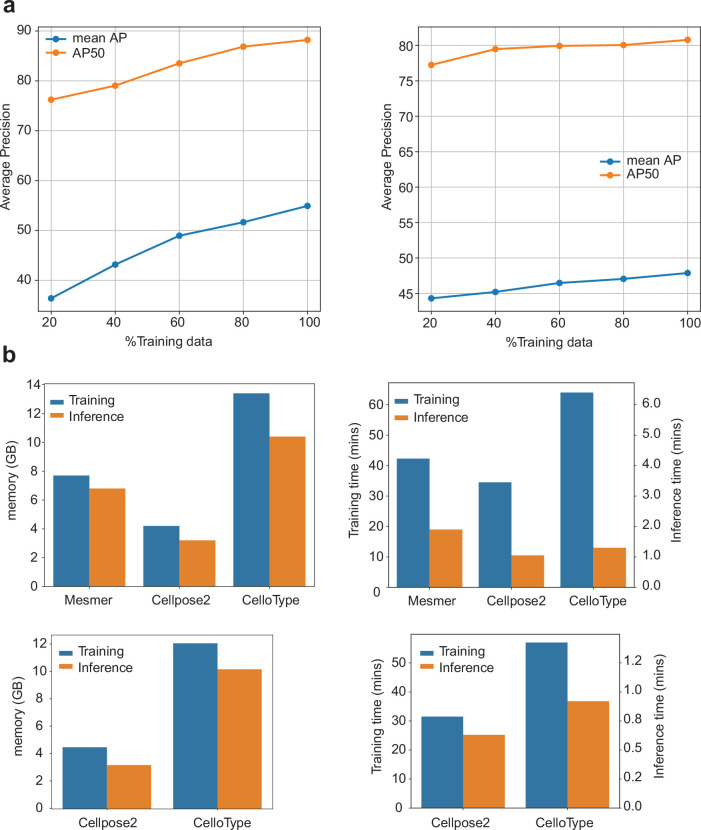
Additional segmentation performance characteristics of compared methods. **a**) CelloType performance using varying amount of training data. AP and AP50 values as a function of the amount of training data. CelloType was trained using various amounts of the training data from the TissueNet (left) and Cellpose Cyto (right) datasets. **b**) Memory usage and running time of the compared methods on the TissueNet (top) and Cellpose Cyto (bottom) datasets. Benchmarking experiments were run using a Dell workstation with an Intel Xeon 6342 CPU (24 cores) and 4 Nvidia A100 GPUs.

Next, we evaluated the segmentation performance of CelloType as a function of the amount of training data. As shown in Extended Data Fig. 2a, even using only 20% of the full TissueNet training set (520 images), CelloType achieved a mean AP of 0.36. As the amount of training data increased, the performance of CelloType progressively improved. For the Cellpose Cyto dataset, CelloType was less sensitive to the amount of training data as the mean AP values were 0.44 and 0.48 using 20% and 100% training data, respectively. In addition, CelloType has speed and memory usage comparable to those of Mesmer and Cellpose2 (Extended Data Fig. 2b).

### Benchmark of cell segmentation on transcript-based images

Imaging-based spatial transcriptomics data represent another major type of spatial omics data. We applied CelloType to datasets generated using two representative spatial transcriptomics platforms: 10x Genomics Xenium and multiplexed error-robust fluorescence in situ hybridization (MERFISH)^27^ assays. These datasets were generated using fresh frozen or formalin-fixed, paraffin-embedded (FFPE) samples from both human and mouse tissues.

**Fig. 4:**
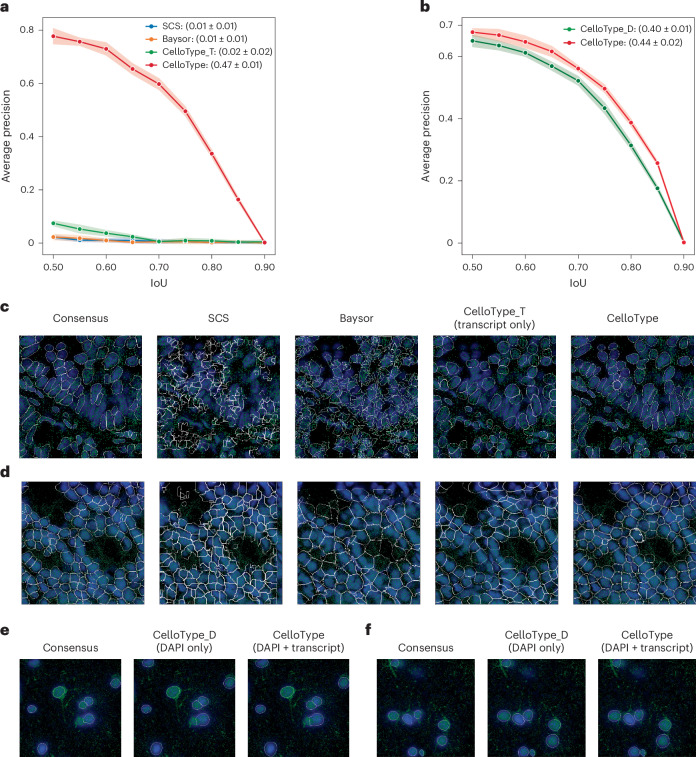
Evaluation of segmentation accuracy on spatial transcriptomics data. **a**, A line plot showing AP across IoU thresholds for cell segmentation using Xenium data by SCS, Baysor, CelloType_T (use transcript signal only) and CelloType (use transcript plus DAPI signals). Each data point represents the average AP from a 10-fold cross-validation experiment. The band width around each line represents the standard deviation. The mean and standard deviation of average AP values across IoU thresholds are shown in the parentheses. **b**, A line plot showing AP values across IoU thresholds for nuclear segmentation using MERFISH data. CelloType_D (use DAPI signal only) and CelloType (use transcript plus DAPI signals). **c**, Representative results of cell segmentation of human lung tissue imaged using Xenium protocol. Blue, nuclear signal (DAPI); green, transcripts signal; white, cell boundary. **d**, Representative results of cell segmentation of mouse colon tissue imaged using the MERFISH protocol. **e**,**f**, Two representative results of nuclear segmentation of human brain tissue imaged using the MERFISH protocol.

We first evaluated CelloType on three Xenium datasets, including human lung cancer, pancreas cancer and mouse normal colon tissues. We constructed ground-truth segmentation masks based on 4′,6-diamidino-2-phenylindole (DAPI) and cell membrane staining signals (Methods) based on the consensus of Mesmer and Cellpose2. Existing state-of-the-art methods for cell segmentation of spatial transcriptomics data, subcellular spatial transcriptomics cell segmentation (SCS)^28^ and Baysor^29^, require both DAPI and transcript signals as the input. In most existing spatial transcriptomics datasets, membrane staining is typically unavailable, which poses challenges for methods such as Mesmer and Cellpose2. Therefore, we used DAPI and transcript signals as the input for SCS, Baysor and CelloType. Based on 10-fold cross-validation, CelloType achieved an average mean AP value of 0.47, compared with 0.01 for both SCS and Baysor (Fig. 4a and Supplementary Table 11). In addition, we evaluated CelloType’s performance using transcript signal as the only input, where it achieved an average mean AP of 0.02. This result suggests that transcript signal alone is insufficient for high segmentation accuracy.

The human MERFISH dataset does not contain membrane staining signal, so we only tested the nuclear segmentation performance of CelloType. Since SCS and Baysor only predict cell boundaries, they were not included in this analysis. We evaluated CelloType using two types of input: DAPI only and DAPI + transcript. As shown in Fig. 4b, based on 10-fold cross-validation, CelloType achieved an average mean AP of 0.44 with the combined input, outperforming CelloType with DAPI signal as the only input that scored an average mean AP of 0.40 (Fig. 4b and Supplementary Table 12). This result suggests that CelloType effectively incorporates transcript signal to enhance nuclear segmentation.

Figure 4c shows a representative result from the human lung cancer FFPE sample, highlighting that the segmentation boundaries produced by SCS and Baysor are not smooth, suggesting poor cell shape priors. Figure 4d shows another representative result from fresh frozen mouse colon sample. The performance of all three methods appears to benefit from the higher transcript density of this sample (compared with the FFPE samples). Figure 4e,f shows representative nuclear segmentation results on the human MERFISH dataset.

### Joint segmentation and classification of multiplexed images

To assess the performance of CelloType for simultaneous cell segmentation and classification, we applied it to a colorectal cancer CODEX dataset^30^. This dataset consists of 140 images of tumor tissue sections from 35 patients. Each tissue section was imaged using 56 fluorescently conjugated antibodies plus two nuclear stains, resulting in a total of 58 channels. These images were processed into 512 × 512 pixel image patches, which were subsequently divided into a training set of 720 patches and a test set of 120 patches. Given the lack of established methods for simultaneous cell segmentation and classification, we combined Cellpose2 and CellSighter as a baseline model. This choice was motivated by the reported superior performance of each method for their respective task (Cellpose2 for segmentation and CellSighter for cell type classification). In addition, we included the Mask R-CNN model^12^ to represent an alternative end-to-end approach, but this has not been applied to cell segmentation.

**Fig. 5:**
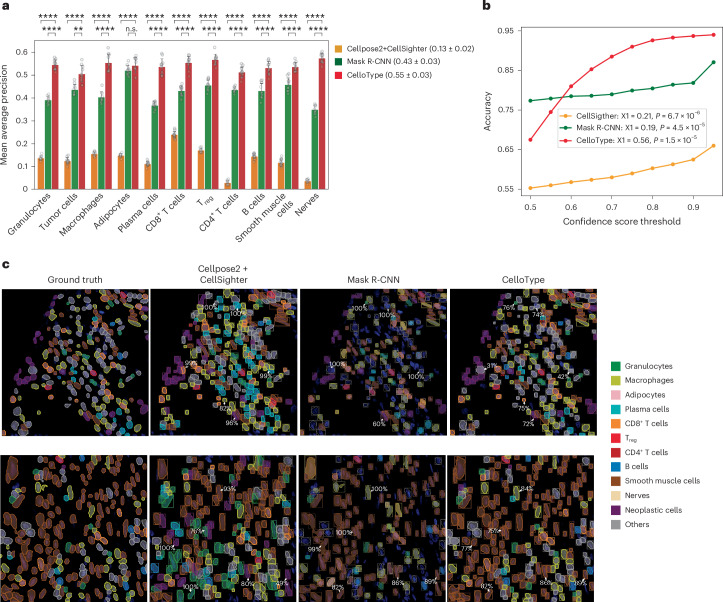
CelloType performs joint segmentation and cell type classification. **a**, A grouped barplot showing mean AP values for cell type predictions by the compared methods. The bar height represents mean values for each cell type from a 10-fold cross-validation experiment. Each grouped barplot is overlaid with ten data points, representing the results of a 10-fold cross-validation. The error bar represents the standard deviation. The average values across cell types are shown in the parentheses. Statistical significance is indicated as follows: ****P < 1 × 10^−4^, ***P < 1 × 10^−3^, **P < 1 × 10^−2^, *P < 0.05. *P* values were computed using one-sided Student’s *t*-test (Supplementary Table 13). **b**, A line plot showing the relationship between classification accuracy and confidence score threshold by the compared methods. X1, fitted coefficient for linear regression between classification accuracy and confidence score; pval, *P* value for the coefficient. **c**, Representative examples of cell segmentation and classification results using the colorectal cancer CODEX dataset. Each row represents a 200 × 200 pixel FOV of a CODEX image. Each FOV shows predicted cell segmentation masks (boxes) and cell types (colors). Ground truth, manually annotated cell types; Cellpose2 + CellSighter, cell segmentation by Cellpose2 followed by cell type classification by CellSighter. Randomly selected confidence scores for cell classification computed by the compared methods are displayed next to the predicted instances.

Using manual cell type annotation as the ground truth, we computed the AP value for each cell type. Based on 10-fold cross-validation, CelloType achieved a mean AP of 0.55 across all cell types, markedly exceeding the Cellpose2 + CellSighter model’s mean AP of 0.13 and the Mask R-CNN model’s mean AP of 0.43 (Fig. 5a and Supplementary Table 13). Furthermore, all three methods produce a confidence score for their cell type predictions. To assess the utility of the confidence score, we explored the relationship between these confidence scores and accuracy of predictions. Notably, CelloType’s confidence scores demonstrated a strong, nearly linear correlation with prediction accuracy, particularly within the confidence score range of 0.5–0.7. In contrast, the relationship for CellSighter’s and Mask R-CNN’s confidence scores appeared flat, indicating a lack of reliable calibration in its confidence assessment (Fig. 5b). Quantitatively, we computed the coefficient of linear regression between prediction accuracy and confidence score threshold. The coefficient for CelloType was 0.56 compared with 0.21 for CellSighter and 0.19 for Mask R-CNN, suggesting that CelloType’s confidence score is more informative.

Figure 5c shows two examples of predictions by Cellpose2 + CellSighter, Mask R-CNN and CelloType, along with the ground-truth annotations. These predictions encompass cell segmentation masks, predicted cell types and associated confidence scores. CelloType correctly predicted the identities of the vast majority of cells of different types with varying morphologies and abundance. For instance, in the top image, CelloType correctly predicted abundant neoplastic cells, alongside rare regulatory T cells (T_reg_) and morphologically irregular macrophages. Similarly, in the bottom image, CelloType correctly predicted abundant smooth muscle cells and sparsely distributed CD8^+^ T cells. In contrast, the Cellpose2 + CellSighter model misclassified several cell types as plasma cells (Fig. 5c, top) and granulocytes (Fig. 5c, bottom). Moreover, we found many instances where CellSighter’s predictions, despite being incorrect, were accompanied by high confidence scores, as indicated by arrows. Mask R-CNN tended to miss some true positive cells, for example, cells next to the highlighted cells.

**Extended Data Fig. 3:**
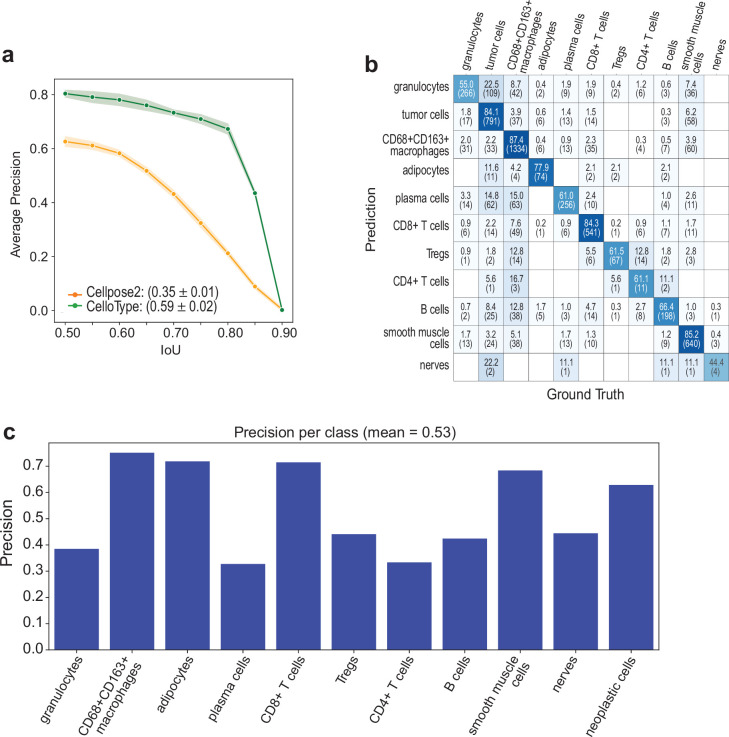
Performance benchmarking of Cellpose2 and CellSighter for separate task. Each method was evaluated for its originally intended task, namely Cellpose2 for segmentation and CellSighter for cell classification. Colorectal cancer CODEX dataset was used for benchmarking purpose. **a**) AP value of segmentation across a range of IoU thresholds. Each data point represents the average AP from a 10-fold cross-validation experiment. Band width around each line represents the standard deviation. Mean and standard deviation of average AP values across IoU thresholds are shown in the parenthesis. **b**) Heatmap showing the confusion matrix of CellSighter cell type classification results. Ground truth cell segmentation masks were used as input to CellSighter. Each grid in the heatmap includes an accuracy score and the count of cells. **c**) Barplot showing the precision for each class identified by the CellSighter model based on the ground truth cell segmentation mask, with an overall mean precision of 0.53.

We next evaluated the performance of each component of the Cellpose2 + CellSighter model, focusing on the segmentation function of Cellpose2 and the cell type classification function of CellSighter. For cell segmentation, Extended Data Fig. 3a shows the AP–IoU curve on the colorectal cancer CODEX dataset. CelloType achieved a mean AP of 0.59 based on 10-fold cross-validation, significantly exceeding Cellpose2’s mean AP of 0.35 (Supplementary Table 14). In assessing CellSighter’s classification performance, we used the ground-truth segmentation masks as the input, treating the task purely a classification task. The resulting confusion matrix revealed the distribution of predictions for each cell type and the accuracy values displayed along the diagonal (Extended Data Fig. 3b). Furthermore, Extended Data Fig. 3c shows CellSighter’s classification precision for 11 cell types, achieving a mean precision of 0.53 (Extended Data Fig. 3c), compared with CelloType’s mean classification precision of 0.81 using an IoU threshold of 0.5. This analysis underscores CelloType’s superior performance not only as an end-to-end tool for cell type annotation but also in its individual functions for segmentation and classification, outperforming the two-stage approach of combining Cellpose2 and CellSighter.

**Extended Data Fig. 4:**
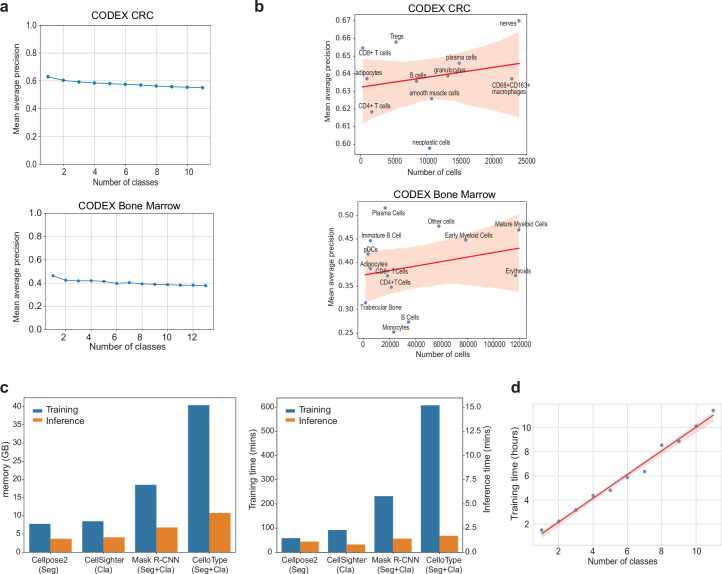
Additional classification performance characteristics of CelloType. **a**) Mean average precision as a function of the number of classes in CODEX CRC (top) and bone marrow (bottom) datasets. **b**) Scatter plot of the number of cells for each cell type and AP performance using the CODEX CRC (top) and bone marrow (bottom) datasets. The linear regression line is shown with the 0.95 confidence interval bands. **c**) Memory usage and running time of the compared methods on the CODEX CRC dataset. **d**) Training time as a function of the number of classes in the CODEX CRC dataset. Seg, segmentation. Cla, classification. The linear regression line is displayed with 0.95 confidence interval bands. Benchmarking experiments were run using a HPC node with an Intel Xeon 6342 CPU (24 cores) and 4 Nvidia A100 GPUs.

We also evaluated the classification performance, speed and memory usage of CelloType as a function of the number of cell types (that is, number of classes) in the image. We performed an analysis where the number of classes was incrementally increased using the CRC CODEX dataset, which has 11 classes. Extended Data Fig. 4a shows that the mean AP value had a small but appreciable decrease from 0.63 with 1 class to 0.55 with 11 classes. This suggests that, while increasing the number of classes makes the problem more challenging, CelloType remains robust as the decrease in performance slows when the number of classes is greater than 10. CelloType was also robust to class imbalance, as the mean AP value did not significantly drop for rare cell types. However, the positive slope of the regression line suggests that CelloType benefits from larger amounts of data (Extended Data Fig. 4b). CelloType has speed and memory usage comparable to those of the Cellpose2 + CellSighter and Mask R-CNN models (Extended Data Fig. 4c,d).

### Multiscale segmentation and annotation by CelloType

Noncellular components, such as the vasculature, lymphatic vessels, trabecular bone and extracellular matrix, and reticular fibers play important roles in tissue function. These elements are typically much larger than cells. Moreover, certain cell types such as macrophages and adipocytes are either large or possess irregular shapes. Together, these elements present challenges to conventional segmentation methods. Furthermore, existing methods are incapable of simultaneous multiscale segmentation of both cellular and noncellular elements within a tissue image. To assess the effectiveness of CelloType for multiscale segmentation and classification, we applied it to a human bone marrow CODEX dataset^31^. This dataset comprises 12 whole-slide images of bone marrow sections from healthy donors, with each tissue section imaged using 53 fluorescently conjugated antibodies plus one nuclear stain, totaling 54 channels. The images were divided into 512 × 512 pixel patches with 1,600 patches for training and 400 patches for testing. The dataset presents a unique challenge due to the diversity of cell/noncell types, notably adipocytes, which are substantially larger than other cell types, and trabecular bone fragments, which have irregular and complex shapes.

**Fig. 6:**
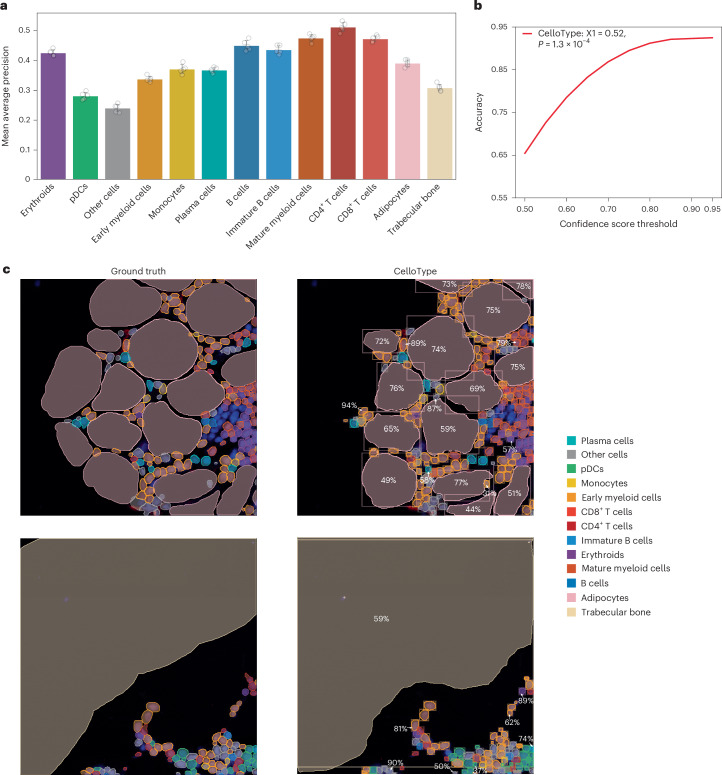
CelloType supports joint multiscale segmentation and classification. **a**, A grouped barplot showing AP stratified by cell and microanatomic structure type. Each grouped barplot is overlaid with five data points, representing the results of a 5-fold cross-validation. The bar height represents mean values from a 5-fold cross-validation experiment. The error bar represents the standard deviation. pDCs, plasmacytoid dendritic cells. **b**, A line plot showing the relationship between classification accuracy and confidence score threshold. X1, fitted coefficient for linear regression between classification accuracy and confidence score; pval, *P* value for the coefficient. **c**, Representative examples of multiscale segmentation and classification using human bone marrow CODEX data. The first row of images shows an example of bone marrow area consisting of various types of smaller hematopoietic cella and much larger adipocytes. The second row of images shows an example of bone marrow area consisting of various hematopoietic cell types and microanatomic structures such as trabecula bone fragments. Randomly selected confidence scores for cell classification are displayed next to the predicted instances.

Using 5-fold cross-validation, we evaluated the performance of CelloType on simultaneous segmentation and classification of both cell and noncell elements in the bone marrow, including small regularly shaped cell types and much larger adipocytes and irregularly shaped trabecular bone fragments. CelloType achieved mean AP values of 0.39, 0.31 and 0.42 for adipocytes, trabecular bone fragments and the rest of cell types, respectively (Fig. 6a). Consistent with our results with the colorectal cancer CODEX dataset, we observed a strong correlation between the prediction confidence scores and prediction accuracy (Fig. 6b). Figure 6c shows two representative examples of predictions by CelloType along with the ground truths. In addition to correctly identifying smaller and regularly shaped cells, CelloType correctly identified most adipocytes and trabecular bone fragments. This result demonstrates CelloType’s efficacy in analyzing challenging tissue images consisting of tightly packed cells and noncell elements with varying sizes and shapes.

## Discussion

We present CelloType, an end-to-end method for joint segmentation and classification for biomedical microscopy images. Unlike existing methods that treat segmentation and classification as separate tasks, CelloType uses a multitask learning approach. By leveraging advancements in transformer-based deep learning techniques, CelloType offers a unified approach to object detection, segmentation and classification. It starts with Swin Transformer-based feature extraction from an image, followed by the DINO object detection and classification module, which produces latent features and detection boxes that, when combined with the raw image inputs within the MaskDINO module, culminate in refined instance segmentation and classification. The shared encoder in the DINO module extracts latent information that is shared by both tasks, explicitly enhancing the connection between the segmentation and classification tasks and simultaneously boosting the performance of both tasks. Moreover, the improved object detection accuracy of DINO through deformable attention and contrastive denoising allows the classification task to focus on relevant regions of the image.

It should be noted that this work has the following limitations. First, CelloType requires training for segmentation and classification tasks. In terms of segmentation, there is a rapid growth of training data, exemplified by resources such as TissueNet and Cellpose Cyto databases. Models that are pretrained on these public datasets are readily transferable to new images, provided that they contain nuclear and/or membrane channels. However, for classification, training data are considerably more limited. As a result, the pretrained CelloType classification model cannot be readily applied to new images unless there is a substantial overlap of cell/structure types between the training and testing images. To mitigate this need for training data for classification, methodologies such as few-shot learning^32^, self-supervised learning and contrastive learning^33^ can be incorporated into the CelloType framework. Specifically, few-shot learning can be incorporated into the CelloType framework by leveraging meta-learning techniques such as prototypical networks^34^ or model-agnostic meta-learning^35^, and contrastive learning can be achieved by adding a contrastive loss function during the training process. In addition, with the rapid growth of spatial omics data, it is anticipated that high-quality tissue annotations will also grow quickly. Consequently, CelloType’s pretraining process can be broadened to include a wider array of datasets, thereby facilitating its application in automated annotation of common tissue types.

Spatial transcriptomics technologies can profile hundreds to thousands of genes at single-cell resolution, yielding a much larger number of features compared with spatial proteomics technologies such as CODEX that typically can only profile fewer than a hundred proteins. This substantial increase in the feature space, coupled with the distinct spatial distribution patterns of RNA transcripts versus proteins, introduces new computational challenges for segmentation and classification. Our results suggest that transcript signal alone is insufficient for accurate segmentation performance even for advanced DNN models such as CelloType. In addition, cell type classification remains challenging and warrants future research.

## Methods

### CelloType

A schematic overview of CelloType is depicted in Fig. 1a. The method consists of three modules: (1) a feature extraction module based on the Transformer DNN model to generate multiscale image features that are used in the DINO and MaskDINO modules; (2) a DINO module for object detection and classification; and (3) a MaskDINO module for segmentation. The resulting latent features and detected bounding boxes are then integrated with the input image in the MaskDINO module to produce instance segmentation results. Both DINO and MaskDINO modules are integrated in a single neural network model for end-to-end learning.

#### Feature extraction module

Multiscale image features are generated using the Swin Transformer^18^ DNN model. Swin Transformer is a hierarchical version of the original transformer model that utilizes shifted window operations for efficient self-attention. It can capture both local and global features, outperforming conventional convolutional networks in modeling complex image data with improved computational efficiency. Here, we use the Swin-L Transformer model pretrained on the COCO instance segmentation dataset^26^.

#### DINO object detection and classification module

The DINO^15^ DNN architecture, standing for ‘DETR with improved denoising anchor boxes’, is a novel end-to-end object detection model improving upon the DETR architecture. DINO leverages the strengths of the Transformer architecture to effectively capture spatial relationships, essential for discerning overlapping or adjacent cells. On the other hand, DINO incorporates denoising techniques essential for the precise identification of cells against intricate backgrounds and under varied imaging conditions. Major components of the DINO architecture in CelloType are described as follows.

##### Query initialization and selection

To generate the initial anchor box for detecting objects, the model uses two types of query: positional queries and content queries. It initializes anchor boxes based only on the positional information of the selected top-*K* features while keeping content queries unchanged. These queries provide spatial information of the objects. Meanwhile, content queries remain learnable and are used to extract content features from the image. This mixed query selection strategy helps the encoder to use better positional information to pool more comprehensive content features, hence more effectively combining spatial and content information for object detection. This mixed query selection method is formulated as

$${Q}_{\text{pos}}={f}_{\text{encoder}}(X\,),{Q}_{\text{content}}=\text{learnable},$$

where *Q*_pos_ and *Q*_content_ represent positional and content queries, respectively. *Q*_pos_ is an *n*-by-4 matrix and *Q*_content_ is an *n*-by-embed_dim matrix where *n* is the number of anchor boxes and embed_dim is the embedding dimension. *X* represents the flattened image features and positional embeddings.

##### Anchor box refinement and contrastive denoising training

DINO refines the anchor boxes step by step across decoder layers using deformable attention^36^. The conventional attention mechanism examines the whole image, whereas the deformable attention selects more important regions of the image and controls the range of self-attention more flexibly, making the computation more efficient. The conventional denoising training technique^37^ involves adding controlled noise to ground-truth labels and boxes, formulated as

$$|\Delta\,x| < \lambda \frac{w}{2},|\Delta y| < \lambda \frac{h}{2},|\Delta w| < \lambda w,|\Delta h| < \lambda h,$$

where (*x*, *y*, *w*, *h*) denote a ground-truth bounding box where (*x*, *y*) are the center coordinates of the box and $w$ and $h$ are the width and height of the box. $\lambda$ denotes a hyperparameter controlling the scale of noise. Contrastive denoising training adds both positive and negative samples of the same ground truth, enhancing the model’s ability to distinguish between objects. DINO involves generating two types of query (positive and negative) with different noise scales *λ*_1_ and *λ*_2_, where *λ*_1_ < *λ*_2_. In this paper, we set *λ*_1_ = 0.4 and *λ*_2_ = 1.

##### Classification head and confidence score

For the classification of each bounding box, a linear transformation is applied to the corresponding denoised features. The linear layer outputs a logit vector $\mathbf{Z}=\left[{z}_{1},{z}_{2},\ldots ,{z}_{K+1}\right]$, where *K* is the number of classes. The vector represents the raw predictions for *K* classes and the ‘no object’ class. Subsequently, a SoftMax function is employed on the logit vector to compute the class probabilities

$${\rm{SoftMax}}\left({z}_{i}\right)=\frac{{{\mathrm{e}}}^{{z}_{i}}}{{\sum }_{j=1}^{K+1}{{\mathrm{e}}}^{{z}_{j}}}.$$

The confidence score for each detected object is taken as the maximum class probability (excluding the ‘no object’ class) output by the model. This score represents the model’s confidence in its prediction of the class for the detected object.

#### MaskDINO segmentation module

We use MaskDINO^17^ to predict the segmentation masks using outputs from the feature extractor module and DINO decoder. MaskDINO enhances the DINO architecture by integrating a mask prediction branch. This mask branch utilizes the DINO decoder’s content query embeddings, ${q}_{{\mathrm{c}}}$, to perform dot-product operations with pixel embedding maps, derived from both image and latent features at high resolution. These operations result in a set of binary masks, where each segmentation mask, $m$, is computed as

$$m={q}_{{\mathrm{c}}}\otimes M\left(T\left({C}_{{\mathrm{b}}}\right)+F\left({C}_{{\mathrm{e}}}\right)\right),$$

where *q*_c_ is the content query embedding, *M* is the segmentation head, *T* is a convolutional layer to map the channel dimension to the transformer hidden dimension, *C*_b_ is the feature map from the feature extractor module, *C*_e_ is the latent features from the DINO transformer encoder, and $F$ is an interpolation-based upsampling function to increase the resolution of latent feature and to make the result match the size of the image feature.

Segmentation task, being a pixel-level classification task, offers more detailed information in the initial training stages compared with the region-level object detection task. Therefore, MaskDINO employs the unified and enhanced query selection technique, which enables the DINO object detection module to leverage the detailed information from the segmentation task early in the training process, enhancing the detection task by providing better-initialized queries for subsequent stages. This cooperative task approach between detection and segmentation results in improved detection performance due to the enhanced box initialization informed by segmentation mask.

During the unified model training, the loss function is calculated by considering three components: segmentation mask, bounding box prediction and class prediction. The composite loss function is expressed as

$$\text{Loss}={\lambda }_{\text{cls}}{L}_{\text{cls}}+{\lambda }_{\text{box}}{L}_{\text{box}}+{\lambda }_{\text{mask}}{L}_{\text{mask}},$$

where *L*_cls_, *L*_box_ and *L*_mask_ represent classification, bounding box and segmentation mask losses, respectively, and *λ*_cls_, *λ*_box_ and *λ*_mask_ are their corresponding weights.

### Implementation of CelloType for segmentation tasks

The CelloType software was implemented using the Detectron2 library. Detectron2 is a Facebook AI Research open-source library that provides a high-performance easy-to-use implementation of state-of-the-art object detection algorithms written with PyTorch^38^. Furthermore, it efficiently manages large datasets and features a flexible architecture that facilitates customization and integration of various image detection or segmentation pipelines.

Dataset was randomly divided into 80% for training, 10% for validation and 10% for testing. All images and cell/nuclear masks in the training, validation and testing sets were converted to align with Detectron2’s JavaScript Object Notation (JSON) dictionary schema. For the training dataset, bounding boxes were derived for each cell using the ground-truth segmentation masks. The final dictionary encompasses the bounding box, segmentation mask and raw image for each cell.

For model training, we initialized the DINO and MaskDINO parameters using the weights pretrained on the COCO instance segmentation dataset, as this dataset is extensive and diverse, providing a foundational knowledge for the model. This pretraining helps to improve feature extraction and generalization. We used the Adam optimizer with a learning rate of 10^−6^ and a batch size of 8. For every five training epochs, the trained model was evaluated on the validation set. The training was terminated when the evaluated AP scores did not improve after 15 epochs.

The model with the best AP scores was used for predicting the cell masks.

For evaluation and testing, we set the number of queries to 1,000, which determines the number of boxes and masks generated by the model. In general, this number should exceed the instance count in each image yet remain reasonable to reduce computational cost. Considering the maximum cell count in an image patch in all our datasets does not exceed 1,000, this number was used as the default parameter. Consequently, the model outputs 1,000 instances per image, each comprising a segmentation mask and a corresponding confidence score. For testing, a confidence threshold of 0.3 was used to call predicted instances.

### Implementation of CelloType for the Xenium dataset

Each Xenium spatial transcriptomics dataset contains three types of signal: DAPI signal, membrane signal and transcript signal. For performance benchmarking, we used the membrane signal only to create the ground-truth cell segmentation masks. DAPI signal and transcript signal were used as the input to the compared methods.

To construct the consensus cell segmentation masks as ground truth, both DAPI and membrane signals were employed. For Mesmer, we used the model pretrained on the TissueNet dataset by the authors of Mesmer. For Cellpose2, we used the model pretrained on the cyto2 dataset by the authors of Cellpose2. The final set of ground-truth cell segmentation was generated by taking the consensus outputs from Mesmer and Cellpose2. Specifically, for each cell, if the IoU of the segmentation results from both methods exceeded 0.3, their intersection was considered the consensus. A total of 4,293 image patches were obtained from human lung, human pancreas and mouse colon tissues. These patches were randomly divided into ten folds for a 10-fold cross-validation experiment. Specifically, it consists of 3,435 images for training, 429 images for evaluation and 429 images for testing.

For training the CelloType model, each image was divided into 512 × 512 pixel patches. For each patch, a transcript density map was computed using kernel density estimate. Subsequently, composite images were generated by overlaying the transcript density map and transcript signal with the DAPI image. In addition, image patches containing only transcript density map and transcript signal were generated to evaluate the performance of CelloType solely on the basis of transcript information.

Key hyperparameters used in CelloType model training included BASE_LR = 0.0001, IMS_PER_BATCH = 16 and MAX_ITER = 20,000.

### Implementation of CelloType for the MERFISH dataset

The MERFISH spatial transcriptomics dataset includes DAPI and transcript signals. For nuclear segmentation benchmarking, we created ground-truth segmentation masks using the DAPI signal. Specifically, we generated the ground truth by combining the outputs of Mesmer (pretrained on the TissueNet dataset) and Cellpose2 (using the built-in ‘model_type=nuclei’) in the same manner as described for the Xenium data analysis.

For training the CelloType model, each field of view (FOV, 2,048 × 2,048 pixels) was divided into 512 × 512 pixel patches. The inputs were composite images of DAPI signal, transcript signal and transcript density map. In addition, we trained a model using only the DAPI signal as the input. We used a total of 700 image patches from the H22 sample. These were randomly divided into ten folds for a 10-fold cross-validation experiment. Specifically, each fold consisted of 560 images for training, 70 images for evaluation and 70 images for testing.

Key hyperparameters for CelloType model training included BASE_LR = 0.0001, IMS_PER_BATCH = 16 and MAX_ITER = 5,000.

### Implementation of CelloType for the classification task

The same training, validation and testing protocols were used as for the segmentation task. However, during model training for multiplexed images with over three channels, the n_channels hyperparameter within the Swin Transformer was set to match the input images’ dimensionality.

### Running of existing methods

#### Mesmer

Mesmer was run using the pretrained model detailed by the authors in the ‘Mesmer-Application.ipynb’ notebook located in the DeepCell-tf GitHub repository. Key parameter settings included image_mpp = 0.5 and compartment = ‘whole-cell’ for cell segmentation and ‘nuclear’ for nuclear segmentation.

#### Cellpose2

For TissueNet and Cellpose Cyto datasets, Cellpose2 was run using the pretrained model provided by the authors.

#### CellSighter

We trained the CellSighter cell type classification model following the protocol provided by the authors. Key parameter settings included crop_input_size = 60, crop_size = 128, epoch_max = 300 epochs and lr = 0.001.

#### Combining Cellpose2 and CellSighter for benchmark

We devised a baseline model combining Cellpose2 and CellSighter, given their reported high performance in the respective tasks. Training of the hybrid model comprised two steps, each optimizing the performance of the individual method. For Cellpose2, CODEX images and corresponding ground-truth cell segmentation masks were used for model training. We retrained the Cellpose2 model following the procedure described by the authors at https://cellpose.readthedocs.io/en/latest/gui.html#training-your-own-cellpose-model. Key parameter settings included weight_decay = 1 × 10^−4^, SGD = True, learning_rate = 0.1 and n_epochs = 100. For CellSighter, the same ground-truth cell segmentation masks along with associated cell type labels were used for training.

During the testing phase, a CODEX image was processed with the trained Cellpose2 model to produce cell segmentation masks, which were subsequently used by the trained CellSighter model for cell type classification. The final results were the combination of the segmentation results of Cellpose2 and cell type classification results of CellSighter.

#### Mask R-CNN

We used the ‘R50-FPN’ model pretrained on the COCO instance segmentation dataset. For training using the CRC CODEX dataset, we used the following parameter settings: BASE_LR = 0.02, IMS_PER_BATCH = 16 and MAX_ITER = 10,000.

#### SCS

SCS was run using the default parameters suggested by the authors at https://github.com/chenhcs/SCS.

#### Baysor

Baysor was run following the instructions by the authors at https://github.com/kharchenkolab/Baysor. For Xenium datasets, key parameters settings included min-molecules-per-gene = 50, min-molecules-per-cell = 30 and scale = 20. For MERFISH datasets, key parameters settings included min-molecules-per-gene = 50, min-molecules-per-cell = 30 and scale = 35.

### Metrics and procedure for evaluating segmentation accuracy

The AP metric is a widely adopted standard for evaluating the performance of instance segmentation methods in computer vision tasks^39,40^. Specifically, for a given IoU threshold, $t$, a prediction is considered a true positive if the IoU between the predicted segmentation and the ground truth is greater than $t$. The IoU is defined as the ratio of the area of overlap between the predicted segmentation mask and the ground-truth mask. The AP is calculated at IoU values from 0.50 to 0.9 with a step size of 0.05. This gives a more comprehensive understanding of a model’s performance, from relatively lenient (IoU 0.50) to stricter overlaps (IoU 0.90).

In the context of multiple classes, mAP is computed by taking the mean of the AP values calculated for each individual class. Specifically, if the task has only one class, such as cell segmentation or nuclear segmentation, the mAP would be the AP across all the IoU thresholds we evaluated. This gives an overall sense of the method’s performance across the various classes in the dataset, rather than focusing on its efficacy in detecting a single class.

To evaluate segmentation performance using the AP metric, we used the COCO evaluation package, a widely used, standardized benchmarking tool in the field of instance segmentation. Segmentation results were first converted into the COCO format before the AP metric was computed using the package. To eliminate redundant detections and ensure that each object is uniquely identified, the package implements the nonmaximum suppression (NMS) procedure. NMS selectively filters out overlapping bounding boxes, retaining only the box with the highest confidence score while discarding others with substantial overlap, as determined by the IoU threshold. Since methods such as Mesmer, Cellpose2 and CelloType do not generate confidence scores for the predicted segmentation masks, we arbitrarily assigned the confidence score to be 1. For CelloType_C and Mask R-CNN that output the confidence score, we used the actual confidence scores computed by the method when applying the NMS procedure.

### Datasets

#### TissueNet dataset

The TissueNet dataset^4^ consists of 2,601 training and 1,249 test multiplexed images collected using multiple imaging platforms and tissue types. Imaging platforms include CODEX, CycIF, IMC, MIBI, MxIF and Vectra. Tissue types include breast, gastrointestinal, immune cells, lung, pancreas and skin. Although many images have dozens of protein markers, all images contain at least two channels necessary for cell/nucleus segmentation: a cell membrane channel and a nuclear channel. Each image contains a manual segmentation of cells and/or nuclei. Each training and test image has a dimension of 512 × 512 pixels and 256 × 256 pixels, respectively.

#### Cellpose Cyto dataset

The Cyto dataset^6^ consists of images from a variety of sources, including the following: (1) cells (Cell Image Library) set: 100 fluorescence images of cultured neurons with both cytoplasmic and nuclear stains obtained from the Cell Image Library database (http://www.cellimagelibrary.org); (2) cells (fluorescence) set: 216 fluorescence images of cells visualized with cytoplasmic markers (this set contains images from BBBC020, BBBC007v1, mouse cortical and hippocampal cells expressing GCaMP6 imaged using a two-photon microscope, confocal images of mouse cortical neurons, and the rest were obtained through Google image search); (3) cells (nonfluorescence) set: 50 bright-field microscopy images from OMERO and Google image search; (4) cells (membrane) set: 58 fluorescence images of cells with membrane maker, 40 of which were from the Micro-Net image set, and the rest were obtained through Google image search; (5) other microscopy set: 86 images of other types of microscopy that contain either noncells or cells with atypical appearances (these images were obtained through Google image search); (6) nonmicroscopy set: 98 images of nonmicroscopy images obtained through Google search of repeating objects including images of fruits, vegetables, artificial materials, fish and reptile scales, starfish, jellyfish, sea urchins, rocks, seashells and so on. All images in the dataset were manually segmented by a human operator.

#### Xenium dataset

We used three publicly available Xenium datasets in this study: normal mouse colon, human lung and pancreas cancer. The Xenium gene panels were designed by 10x Genomics and consist of probes targeting 377 (human panel) and 379 (mouse panel) genes, respectively. Each dataset consists of DAPI staining data, cell membrane staining data and transcript measurement data.

#### MERFISH dataset

The dataset was generated using human superior temporal gyrus tissue samples^27^. The gene panel consists of probes targeting 4,000 genes. The dataset consists of DAPI staining data and transcript measurement data.

#### Colorectal cancer CODEX dataset

This dataset contains CODEX images of 140 human colorectal samples stained with a 56 fluorescently conjugated antibodies and 2 nuclear stains^30^. Cells were segmented using Mesmer. Cell types were annotated by the authors using a combination of iterative clustering and manual examination of marker expression profiles and cell morphology. For each tissue image in the dataset, image patches of 512 × 512 pixels were generated.

#### Bone marrow CODEX dataset

This dataset contains CODEX images of 12 human bone marrow samples stained with 54 fluorescently conjugated antibodies and one nuclear stain^31^. Hematopoietic cell types were annotated by the authors using a combination of iterative clustering and manual examination of marker expression profiles and cell morphology. Due to their large sizes and irregular shapes, adipocytes and trabecular bone fragments were completely manually annotated by the authors.

### Reporting summary

Further information on research design is available in the Nature Portfolio Reporting Summary linked to this article.

## Online content

Any methods, additional references, Nature Portfolio reporting summaries, source data, extended data, supplementary information, acknowledgements, peer review information; details of author contributions and competing interests; and statements of data and code availability are available at 10.1038/s41592-024-02513-1.

## Supplementary information

Reporting Summary

**Supplementary Table 1** **List of** ***P*** **values for comparing CelloType with other methods in AP value at each IoU threshold in the TissueNet cell segmentation task**. One-sided Student’s *t*-test was used for *P*-value calculation, comparing APs from the 10-fold cross-validation results for each IoU threshold. Each row represents an IoU threshold, and each column corresponds to a method being compared with CelloType_C. **Supplementary Table 2** **List of** ***P*** **values for comparing CelloType with other methods in AP value at each IoU threshold in the TissueNet nuclear segmentation task**. One-sided Student’s *t*-test was used for *P*-value calculation, comparing APs from the 10-fold cross-validation results for each IoU threshold. Each row represents an IoU threshold, and each column corresponds to a method being compared with CelloType_C. **Supplementary Table 3** **List of** ***P*** **values for Extended Data Fig. 1a (left barplot)**. One-sided Student’s *t*-test was used for *P*-value calculation, comparing mean APs for the image-level mean AP. **Supplementary Table 4** **List of** ***P*** **values for Extended Data Fig. 1a (right barplot)**. One-sided Student’s *t*-test was used for *P*-value calculation, comparing mean APs from the 10-fold cross-validation results. **Supplementary Table 5** **List of** ***P*** **values for Fig. 2c (top left barplot)**. One-sided Student’s *t*-test was used for *P*-value calculation, comparing mean APs from the 10-fold cross-validation results. **Supplementary Table 6** **List of** ***P*** **values for Fig. 2c (top right barplot)**. One-sided Student’s *t*-test was used for *P*-value calculation, comparing mean APs from the 10-fold cross-validation results. **Supplementary Table 7** **List of** ***P*** **values for Fig. 2c (bottom left barplot)**. One-sided Student’s *t*-test was used for *P*-value calculation, comparing mean APs from the 10-fold cross-validation results. **Supplementary Table 8** **List of** ***P*** **values for Fig. 2c (bottom right barplot)**. One-sided Student’s *t*-test was used for *P*-value calculation, comparing mean APs from the 10-fold cross-validation results. **Supplementary Table 9** **List of** ***P*** **values for comparing CelloType with other methods in AP value at each IoU threshold in the Cellpose Cyto cell segmentation task**. One-sided Student’s *t*-test was used for *P*-value calculation, comparing APs from the 10-fold cross-validation results for each IoU threshold. Each row represents an IoU threshold, and each column corresponds to a method being compared with CelloType_C. **Supplementary Table 10** **List of** ***P*** **values for Fig. 3b**. One-sided Student’s *t*-test was used for *P*-value calculation, comparing mean APs from the 10-fold cross-validation results. **Supplementary Table 11** **List of** ***P*** **values for comparing CelloType with other methods in AP value at each IoU threshold in the Xenium cell segmentation task**. One-sided Student’s *t*-test was used for *P*-value calculation, comparing APs from the 10-fold cross-validation results for each IoU threshold. Each row represents an IoU threshold, and each column corresponds to a method being compared with CelloType. **Supplementary Table 12** **List of** ***P*** **values for comparing CelloType with other methods in AP value for each IoU threshold in the MERFISH nuclear segmentation task**. One-sided Student’s *t*-test was used for *P*-value calculation, comparing APs from the 10-fold cross-validation results for each IoU threshold. Each row represents an IoU threshold, and each column corresponds to a method being compared with CelloType. **Supplementary Table 13** **List of** ***P*** **values for Fig. 5a**. One-sided Student’s *t*-test was used for *P*-value calculation, comparing mean APs from the 10-fold cross-validation results. **Supplementary Table 14** **List of** ***P*** **values for comparing CelloType with Cellpose2 in AP value at each IoU threshold in the CRC CODEX cell segmentation task**. One-sided Student’s *t*-test was used for *P*-value calculation, comparing APs from the 10-fold cross-validation results for each IoU threshold. Each row represents an IoU threshold, and each column corresponds to a method being compared with CelloType.

## Data Availability

The TissueNet dataset is available at https://datasets.deepcell.org/, the Cellpose dataset at https://www.cellpose.org/dataset, the CODEX colorectal dataset at 10.7937/tcia.2020.fqn0-0326, the Xenium dataset at https://www.10xgenomics.com/datasets and the MERFISH Brain dataset at https://datadryad.org/stash/dataset/doi:10.5061/dryad.x3ffbg7mw.

## Code availability

CelloType is available via GitHub at https://github.com/tanlabcode/CelloType.
